# Two-part silicone mold. A new tool for flexible ureteroscopy surgical training

**DOI:** 10.1590/S1677-5538.IBJU.2014.0638

**Published:** 2016

**Authors:** Bruno Marroig, Marco Antonio Q. R. Fortes, Marco Pereira-Sampaio, Francisco J. B. Sampaio, Luciano A. Favorito

**Affiliations:** 1 Departamento de Cirurgia Geral da Universidade do Estado do Rio de Janeiro - UERJ, RJ, Brasil; 2 Departamento de Cirurgia Geral do Hospital Naval Marcílio Dias - HNMD, RJ, Brasil; 3 Urogenital Unidade de Pesquisa da Universidade do Estado do Rio de Janeiro - UERJ, RJ, Brasil

## Abstract

**Introduction and objectives:**

Flexible ureteroscopy is a common procedure nowadays. Most of the training programs use virtual reality simulators. The aim of this study was to standardize the building of a three-dimensional silicone mold (cavity) of the collecting system, on the basis of polyester resin endocasts, which can be used in surgical training programs.

**Materials and Methods:**

A yellow polyester resin was injected into the ureter to fill the collecting system of 24 cadaveric fresh human kidneys. After setting off the resin, the kidneys were immersed in hydrochloric acid until total corrosion of the organic matter was achieved and the collecting system endocasts obtained. The endocasts were used to prepare white color two-part silicone molds, which after endocasts withdrawn, enabled a ureteroscope insertion into the collecting system molds (cavities). Also, the minor calices were painted with different colors in order to map the access to the different caliceal groups. The cost of the materials used in the molds is $30.00 and two days are needed to build them.

**Results:**

Flexible ureteroscope could be inserted into all molds and the entire collecting system could be examined. Since some anatomical features, as infundular length, acute angle, and perpendicular minor calices may difficult the access to some minor calices, especially in the lower caliceal group, surgical training in models leads to better surgical results.

**Conclusions:**

The two-part silicone mold is feasible, cheap and allows its use for flexible ureteroscopy surgical training.

## ARTICLE INFO

**Available at:** **www.intbrazjurol.com.br/video-section/marroig_850_851/**

**Int Braz J urol. 2016; 42 (video #6): 850-1**
